# Adenoid cystic carcinoma of the trachea: a clinico-pathological analysis

**DOI:** 10.11604/pamj.2015.20.240.3953

**Published:** 2015-03-13

**Authors:** Abderrahim Elktaibi, Massine Elhammoumi, Adil Boudhas, Adil Arsalane, Fayçal Eloueriachi, Mohamed Oukabli, Elhassane Kabiri, Abderrahmanne AL Bouzidi

**Affiliations:** 1 Department of Pathological Anatomy, Mohammed V Military Teaching Hospital, Rabat, Morocco; 2 Department of Thoracic Surgery, Mohammed V Military Teaching Hospital, Rabat, Morocco

**Keywords:** Adenoid cystic carcinoma, primary malignant tumor, trachea

## Abstract

Primary malignant tracheal tumors are not common and adenoid cystic carcinoma (ACC) of trachea is very rare. The diagnosis is often delayed due to the atypical symptoms. We report an extremely rare case of ACC of proximal trachea, in a 55-year-old female who presented with a 12 month history of progressive dyspnea. Laryngoscopy and computed tomography revealed a broad-based polypoidal mass arising from posterior wall of the proximal trachea. Biopsy confirmed the diagnosis of ACC. The patient underwent a complete surgical resection and post operative radiotherapy. Six months follow-up of the patient did not reveal local recurrence or distant metastases. The literature of tracheal ACC is reviewed.

## Introduction

Primary tracheal tumors are rare, constitute only 2% of all respiratory tract tumors [1] and representing less than 0.1% of cancer death. Adenoid cystic carcinomas (ACC) are the second most common primary malignant tracheal neoplasms after squamous cell carcinoma [2]. They mostly arise from salivary gland, especially minor salivary glands. They can also arise in various sites of the head and neck including the tracheobronchial tree and even in sites outside the head and neck, such as breast, uterine cervix, prostate, and the skin [3]. The clinical and pathologic features of ACC of the trachea were initially reported in 1859 by Billroth. Complete surgical resection offers the patient a better opportunity of prolonged survival or complete remission [4]. We report a case with adenoid cystic carcinoma of the trachea and discuss epidemiology, pathological features, prognostic factors and therapeutic options of this disease.

## Patient and observation

**Figure 1:**
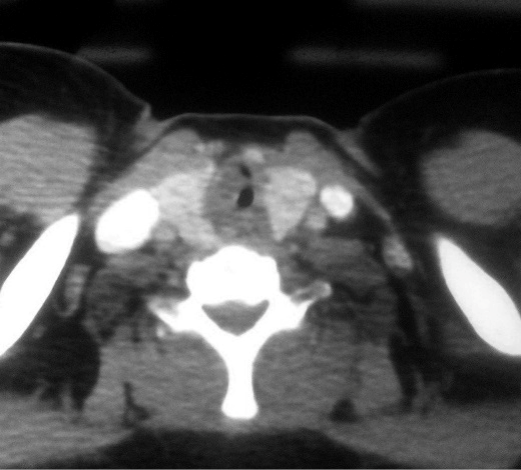
Contrast-enhanced axial computed tomography (CT) scan of neck shows a broad-based soft tissue mass arising from posterior and right side wall wall of trachea causing near total luminal narrowing and having both intraluminal and extraluminal components

**Figure 2:**
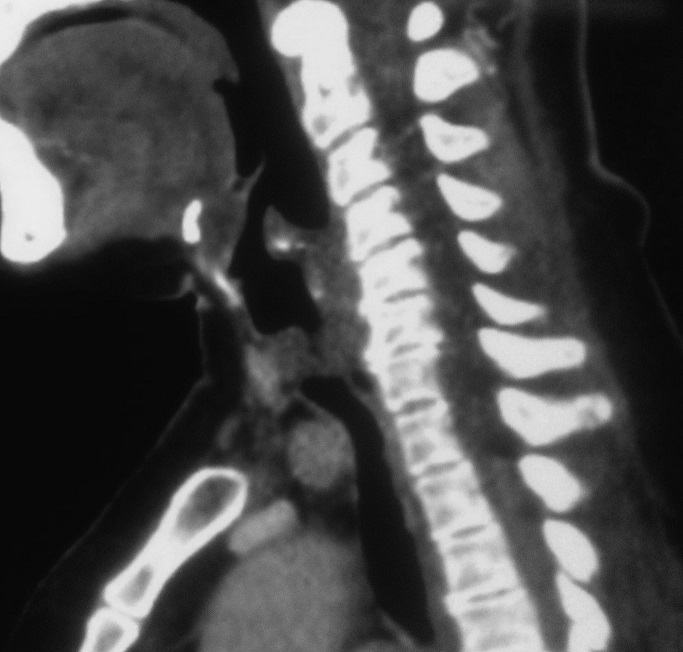
Sagittal reformatted CT images of neck show longitudinal extent of tumor located at upper end of trachea

**Figure 3:**
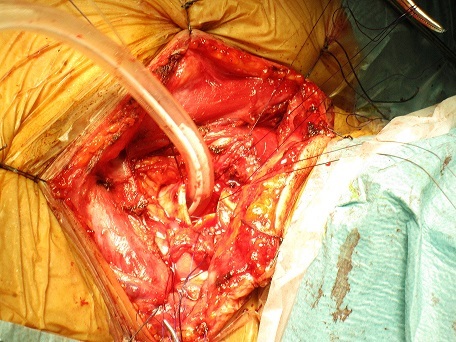
Intraoperative appearance of the tracheal resection with intraluminal tumor

**Figure 4:**
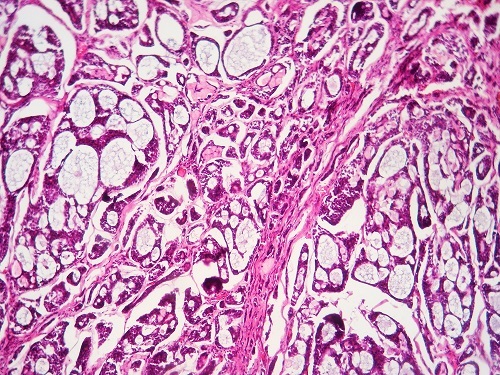
Photomicrograph showing histological features of adenoid cystic carcinoma with uniform hyper chromatic basaloid cells arranged in typical cribriform pattern and surrounding acellular spaces containing mucoid and hyaline material (Hematoxylin-eosin staining ×250)

A 55-year-old female presented with progressive dyspnea and shortness of breath for one year. She experienced malaise in the neck, but had no dysphagia, and sleep bad in supine position at night. She was diagnosed first with asthma. On physical examination, vital signs were normal, trachea was in the midline. On auscultation, there were inspiratory dry rales on laryngeal and both lung fields. Laboratory data were significant for arterial blood gas analysis with partial pressure of oxygen in artery (PaO_2_) 93.1 mmHg, partial pressure of carbon dioxide in artery (PaCO_2_) 56.1 mmHg, arterial oxygen saturation (SaO_2_) 96%. White blood cell (WBC) was 14.10^3^/L. Pulmonary function test showed forced vital capacity (FVC) of 1.9 L (80.6% of predicted), forced expiratory volume in one second (FEV_1_) of 1.74 L (88.3% of predicted). Fibrolaryngoscopy showed bilateral vocal cords were smooth and glottis was normal. Bronchoscopic examination showed an obvious obstruction in cervical trachea with a diameter of 1 cm in the posterior wall of the subglottic trachea, which was smooth and obstructed about 75% of the trachea. The sagittal and coronal reconstruction CT scan of trachea showed a soft tissue image in subglottic trachea (Figure 1, Figure 2). Distant tumor extension was negative. Pathological results from biopsy of tumor revealed cystic adenoid carcinoma. At last a tracheal tumor removal surgery and reconstruction was performed under general anesthesia, and showed the tumor with size of 2 cm × 1 cm was located between arch of first and fourth tracheal ring (Figure 3). Histopathological examination of the surgical specimen confirmed the diagnosis of ACC with negative limits of resection. It showed a cribriform feature, characterized by nests of cells with cylindromatous microcystic spaces. These are filled with hyaline and basophilic mucoid material. The stroma within the tumour is hyalinized. Perineural invasion is observed (Figure 4). Post operative course and bronchoscopic examination were satisfactory six months later.

## Discussion

The incidence of primary tracheal cancer is approximately 0.1 in per 100 000 people per year. Trachea tumors, especially in their early phase, are easily misdiagnosed as asthma due to the similar clinical manifestations between these two diseases [5]. The patient in this report has recurrent episodes of dyspnea and shortness of breath. In contrast to tracheal squamous cell carcinoma (SCC) that occur in men approximately 90% of the time, primary tracheal ACC is found in men and women with almost equal frequency.

The results of a previous study of 174 patients with tracheal ACC showed a female-to-male ratio of 1.1:1.0 (90 women and 84 men). The ages reported for tracheal ACC ranged from 45 to 60 years. Patients with ACC usually present with symptoms such as coughing, wheezing and dyspnea and are often treated for asthma for months to years before being correctly diagnosed. Few patients presented with hoarseness and weight loss [4]. ACC arises more commonly in the minor salivary glands and in the seromucinous glands of the upper respiratory tract. Tracheal tumors mostly arise in the lower or upper third, with a tendency to originate at the lateral and posterolateral wall near the junction of the cartilaginous and membranous portions [1]. In 44 previously reported patients with tracheal ACC, the location was 45.5% (n = 20) in the upper part of the trachea, 20.5% (n= 9) in the middle part of the trachea, and 34% (n =15) in the lower part of the trachea [4]. If there is suspicion of upper airway obstruction, CT scans of trachea are required. The CT scan is a useful imaging procedure for ACC. It is highly accurate in the assessment of the tumor location, extra luminal extensions, carinal involvement and distant metastasis [1]. With the use of helical CT data sets, multiplannar reconstructions have been shown to facilitate the assessment of patients with airways disease and are known to provide various advantages in terms of image quality. The reconstruction images help to assess both the intra and extra luminal growth of the tumor and its longitudinal extent along the tracheal or bronchial wall by allowing the evaluation of extra luminal surrounding tissues [3].

Therefore, helical CT provides precise information about the extent of a tumor, which is important for planning a surgical resection. Unfortunately, this investigation could not be done in our case [1]. Bronchoscopy is the best selection for diagnosing tracheal tumor, can identify the location and nature of tumor. It needs to be emphasized that bronchoscopist should pay close attention to the subglottic trachea, which is usually neglected during bronchoscopy [5]. Pulmonary function testing is indispensable for diagnosis of tracheal tumor. A fixed airway obstruction will result in a decreased inspiratory and peak expiratory flow (PEF). Pathologically these tumors characteristically grow into the airway lumen, forming a smooth surfaced, somewhat polypoid tumor; occasionally, growth is circumferential and annular. Submucosal extension, sometimes to a considerable distance from main tumor is not uncommon. Histologically, three patterns are seen; trabecular, cribriform and solid type. The cribriform pattern is most common consisting of uniform cells with relatively little cytoplasm arranged in well-defined nests of variable size. The cells in these nests are separated by well-defined cystic spaces containing a mucinous substance that stains strongly with alcian blue and weakly with Periodic Acid– Schiff (PAS) [1]. ACC spreads most commonly by direct extension, submucosal or perineural invasion, or hematogenous metastasis. More than 50% of patients with tracheal ACC have hematogenous metastases. Pulmonary metastases are the most common and can remain asymptomatic for many years. Metastases to the brain, bone, liver, kidney, skin, abdomen, and heart have also been reported. Local recurrence of tracheal ACC is common and occurs at an average of 51 months after the primary treatment [4]. Treatment options include surgery alone, radiation therapy alone, or a combination.

The surgical methods are primary tracheal resection and reconstruction, primary tumor resection, and endoscopic resection, either by coring or using a laser. Radiation therapy for ACC of salivary glands has been found to provide improved local control of tumors but did not affect survival. This treatment has not been investigated for tracheal ACC. The role of post-operative adjuvant radiotherapy remains uncertain. It is reasonable to assume that adjuvant radiation therapy may be beneficial and likely delays or reduces the incidence of local recurrence in the airway if surgical removal was complete. According to the results of previous studies the 5-year survival ranged from 66% to 100% and the 10-year survival ranged from 51% to 62% for patients with tracheal ACC regardless of the treatment [4].

## Conclusion

In summary, ACC is a rare primary tracheal malignancy. Hemoptysis, nonproductive cough, and dyspnea are the common initial and atypical symptoms. Complete surgical resection provides the patient with the best chance of prolonged survival or even complete remission. Post-operative radiotherapy may have some effect on local control but did not affect survival rate.
